# A comparative study between Ki67 positive versus Ki67 negative females with breast cancer: Cross sectional study

**DOI:** 10.1016/j.amsu.2020.10.049

**Published:** 2020-10-29

**Authors:** Mowafak Masoud Bahaddin

**Affiliations:** Department of Surgery, College of Medicine, University of Duhok, Duhok, Kurdistan Region, Iraq

**Keywords:** Breast cancer, Ki67, Hormone positive breast cancer, TNM staging System

## Abstract

**Introduction:**

The prognosis of breast cancer depends on several clinical and pathological parameters most importantly the clinical stage, other factors predicting the outcome are hormone receptors like estrogen and progesterone receptors. Expression of Ki67 also have been shown to affect the outcome.

**Patients and methods:**

This retrospective study included 278 female patients diagnosed and operated for breast cancer. Patients were grouped into 2 groups according to the expression of Ki67 to those with positive and those with negative expression. Both groups were compared for differences.

**Results:**

The mean age was 48.61 years and the right breast was the commonest affected side, the mean tumor size was 34 mm, 70% had axillary LN involvement, 50% had intermediate tumor grade, and 85.6% had no recurrence. Most patients had stage IIA, IIB, and IIIA, 67.6% had positive expression of Ki67 and had a significant correlation with the tumor grade, tumor necrosis, and ER expression (P values 0.001, 0.047, and 0.002) respectively, while the correlation was negative with recurrence, axillary LN involvement, TNM stage, site of the tumor, age, tumor size, PR and HER-2 receptor (P values 0.476, 0.971, 0.509, 0.405, 0.122, 0.994, 0.892, and 0.418) respectively.

**Conclusion:**

Most patients with breast cancer have positive expression of Ki67 which has a positive correlation with tumor grade, the presense of necrosis inside the tumor and estrogene receptor status. This marker is directly related with higher degrees of tumor agressiveness and may be useful in modulating different treatment modalities.

## Highlights

• Breast cancer patients have great variability in the biological behavior and cancer aggressiveness.
• Ki67 expression have been shown to affect the outcome of breast cancer patients.
• The gene coding for Ki67 is located on the long arm of chromosome number 10.
• Ki67 is directly related with higher degrees of tumor agressiveness.
• Ki67 may be useful in modulating different treatment modalities.

## 1 Introduction

Breast cancer is the most common type of cancer that affect females during their lifetime, its incidence is increasing worldwide. Breast cancer has various histological types and they differ greatly in the expression of markers based on many genetic factor related to tumor cells [[1], [2], [3], [4]].

Tumors of the same histological types many have great variability in the biological behavior and the degree of aggressiveness, this is due to many tumor and patient factors such as the age, the clinical stage, the type of management and the expression of various markers on the tumor cells [1].

The prognosis of breast cancer depends on several clinical and pathological parameters, the most important one is the clinical stage at diagnosis. The most widely adopted staging system is the Tumor-Node-Metastasis staging system (TNM). Early stages of breast cancer had better outcomes than advanced stages. There are some other factors that play an important role in the prediction of breast cancer outcome like the expression of hormone receptors particularly estrogen and progesterone receptors. As part of these factors the expression of Ki67 marker on the breast cancer cells have been shown to affect the outcome of such patients [3,[5], [6], [7], [8], [9]].

The gene coding for the Ki67 is located on the long arm of chromosome number 10. Ki67 is one of the proteins that regulate cell cycle, it normally reacts with a nuclear non-histone protein which is expressed in all active phases of the cell cycle division, except in the G0, its expression is variable throughout the cell cycle being low during the G1 and the early S phase and being highest during mitosis, a sharp decline occur in the anaphase and telophase. Ki67 is expressed also in normal breast tissues but to lower extent, it is estimated that normal breast tissues express less than 3% of this marker [5,10].

The assessment of Ki67 routinely for all cases of breast cancer is not recommended in most population based studies and meta-analyses, but the most widely accepted recommendation is that a standard framework for the scoring of Ki67 expression must be done by pathologists communicating with the multidisciplinary team for the breast cancer patients [11,12].

Significant improvement occurred in both the diagnosis and the management of breast cancer in the last decades, this is due to population based early detection programs, advancement in the imaging modalities, and the detection of various biological and hormonal factors that are expressed by the tumor and have a direct effect on both the prognosis and the response to various management lines, and the methods of the management should be standardized and objective [3,13].

The aim of this study is to detect any significant correlation between Ki67 expression and different patient and tumor related factor in patients with breast cancer.

## 2 Patients and methods

This is a retrospective study that included 278 female patients who were diagnosed and operated for breast cancer. All females underwent modified radical mastectomy and then the samples were sent for histopathological examination and immunohistochemical analyses. Patients were grouped into 2 groups according to the expression of Ki67 on the tumor tissue, the first group were those who had positive expression for the Ki67 and the other group were those with the negative expression for Ki67. These two groups were compared to detect any difference regarding different tumor and patient's characteristics.

The staging of the breast cancer were done adopting the 8th American Joint Committee on Cancer (AJCC) criteria. Histological grade for the tumor was done according to the modified Scarff-Bloom-Richardson Scoring System. Tumors which express Ki67 al levels less than 14% were regarded negative and those which express 14% or above were regarded positive [2].

An informed consents were obtained from all the participants to be included in this study. In this study we included female patients who were diagnosed with invasive ductal carcinoma with different clinical stages. We excluded male patients and patients with histological types of breast cancer other than invasive ductal carcinoma, patients who refused to be included in this study and those with no sufficient data also were excluded.

## 3 Statistical analyses

Data were described using frequency and percentage for the categorical variables and mean and standard deviation for the continuous ones, tumor factors were displayed according to different main categories and subcategories. The two group of patients according to Ki67 expressions were described and correlations were displayed with various patient and tumor characteristics adopting the Pearson Chi-Square test and the Fisher's Exact test for the categorical variables and the independent *t*-test for the numerical ones.

Significant associations were considered when the P-value was less than 0.05. Data analyses were done using the Statistical Package for Social Sciences (SPSS 24:00 IBM: USA).

## 4 Ethical approval

The research is registered according the World Medical Association's Declaration of Helsinki 2013 at the research registry at the 22nd of September 2020, Research registry UIN: research registry **6041**.

The work of this article has been reported in line with the STROCSS criteria [14].

## 5 Results

**Fig. 1:**
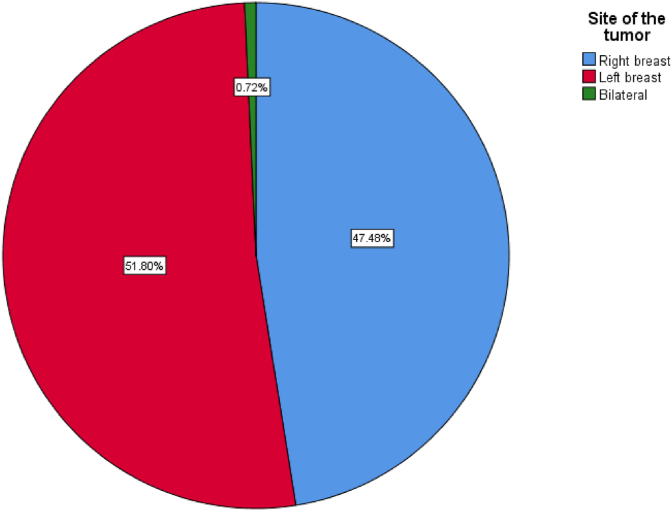
A simple pie chart showing the site of the tumor.

The mean age of our patients was 48.61 years (SD: 11.646), and the most common site of the tumor is the right breast, Fig. 1.

The mean size of the tumor was 34.08 mm and most patients had a positive axillary involvement. Most tumors have intermediate grade, tumor necrosis with no recurrence, Table 1.

**Table 1:** Showing different tumor characteristics.

Tumor characteristics	Subcategories	Frequency	Percentage
Tumor size (M; SD) **Range 5**–**100**		34.08	16.769
Axillary LN status	Positive Negative	195 83	70.1 29.9
Axillary LN (M; SD) **Range: 0**–**31**		3.93	5.467
Grade of the tumor	Low grade tumor Intermediate grade High grade	7 139 132	2.5 50.0 47.5
Tumor necrosis *	Absent Present	87 102	31.3 36.7
Recurrence and/or metastasis	No recurrence Recurrence or metastasis	238 40	85.6 14.4
*In 89 patients (32%) no data were available regarding tumor necrosis			

The majority of tumors were stage IIB, IIIA, and IIA respectively, Table 2.

**Fig. 2:**
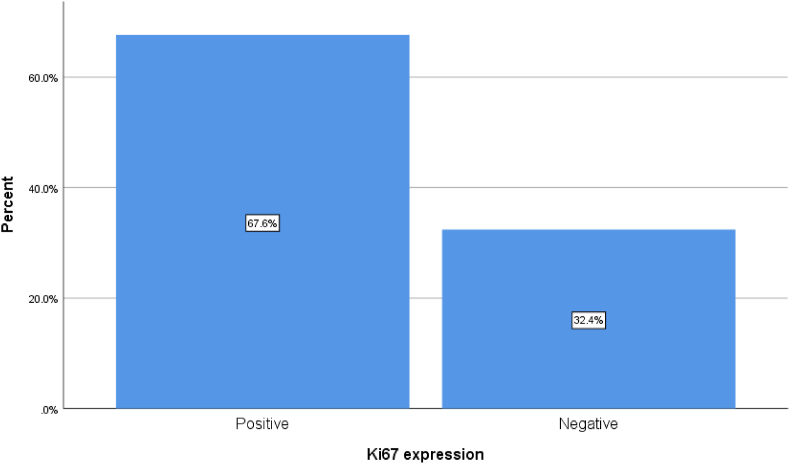
A simple bar chart showing the percentage of both groups.

Most patients have positive expression of KI67, and the expression of other hormone receptors is displayed in Fig. 2 and Table 3.

**Table 2:** Showing the TNM stages of the tumors of the patients involved in this study.

TNM stage	Frequency	Percent
Stage IA	24	8.6
Stage IB	8	2.9
Stage IIA	57	20.5
Stage IIB	71	25.5
Stage IIIA	62	22.3
Stage IIIB	9	3.2
Stage IIIC	28	10.1
Stage IV	19	6.8

**Table 3:** Showing the expression of Ki67 and other hormones on the breast cancer tissue.

Marker/hormone receptor	Mean	Standard deviation	Range
Ki67	29.33	22.615	0–100
Estrogen receptor	48.79	41.284	0–100
Progesterone receptor	40.51	39.410	0–100
HER-2	1.53	1.199	0–5

The two group of patients according to Ki67 expressions were described and correlations were displayed with various patient and tumor characteristics adopting the Pearson Chi-Square test and the Fisher's Exact test for the categorical variables and the independent *t*-test for the numerical ones, Table 4, Table 5.

**Table 4:** Showing the correlation between the KI67 expression and the categorical variables using the cross tabulation.

	Ki67 expression status		Sig.
	Positive	Negative	
Tumor recurrence **No recurrence** **Recurrence and/or metastasis**	159 (66.8%) 29 (72.5%)	79 (33.2%) 11 (27.5%)	0.476*
Axillary lymph node status **Negative** **Positive**	56 (67.5%) 132 (67.7%)	27 (32.5%) 63 (32.3%)	0.971*
Grade of the tumor **Low grade** **Intermediate grade** **High grade**	3 (42.9%) 82 (59.0%) 103 (78.0%)	4 (57.1%) 57 (41.0%) 29 (22.0%)	0.001**
TNM stage **IA** **IB** **IIA** **IIB** **IIIA** **IIIB** **IIIC** **IV**	14 (58.3%) 7 (87.5%) 35 (61.4%) 45 (63.4%) 45 (72.6%) 6 (66.7%) 22 (78.6%) 14 (73.7%)	10 (41.7%) 1 (12.5%) 22 (38.6%) 26 (36.6%) 17 (27.4%) 3 (33.3%) 6 (21.4%) 5 (26.3%)	0.509**
Necrosis inside the tumor **Present** **Absent**	79 (77.5%) 56 (64.4%)	23 (22.5%) 31 (35.6%)	0.047*
Site of the tumor **Right** **Left** **Bilateral**	85 (64.4%) 101 (70.1%) 2 (100.0%)	47 (35.6%) 43 (29.9%) 0 (0.0%)	0.405**
* Pearson Chi-Square test. ** Fisher's Exact test.

**Table 5:** Showing the correlation between the Ki67 expression and the numerical variables using the independent *t*-test.

	Mean Difference	Std. Error Difference	95% Confidence Interval of the Difference		Sig.
			Lower	Upper	
Age at diagnosis	.937	1.494	−2.005	3.879	.122
Tumor size (mm)	.889	2.153	−3.349	5.127	.994
Estrogen receptor	−15.041	5.224	−25.324	−4.758	.002
Progesterone receptor	−19.102	4.952	−28.850	−9.354	.892
HER-2 receptor	.413	.152	.113	.712	.418

## 6 Discussion

Breast cancer is composed of a heterogeneous types of tumors and they differ in regard to the prognosis and the management. Most cases are diagnosed adopting the triple assessment which include the history and clinical examination, imaging and the histopathological examination. The choice of the treatment whether surgical, adjuvant or neoadjuvant one depend on the age of patient, the clinical stage of the tumor, the hormone recerprot status, and HER2 status [[15], [16], [17]].

The association of the Ki67 and braest cancer prognosis is still a matter of great debate, many articles studies this correlation with various conclusions, and most agree that this debate is still open and more studies are still done regarding this subject, a meta-analysis study was done which included 64,196 breast cancer patients, the authors studied the cut off of the level of Ki67 which is associated with high fatality rate, they concluded that the cut off level of 25% was associated with higher fatality rate then patients with lower levels [7,[18], [19], [20]].

One of the interesting findings was that the absence of ki67 expression in normal breast tissue which express ER, which mean that only ER negative cells are proliferating in breast tissue. This feature is lost in breast cancer tissue in which both markers. i.e; Ki67 and ER are expressed in high concentrations [10].

The association between Ki67 and the response to chemotherapy is well studied and most authors agree that it predicts a better response to chemotherapy, higher scores of Ki67 is associated with better response to chemotherapy [15].

Breast cancer with high expression of Ki67 is found to have a worst outcome, in our study the most patients have a positive expression of Ki67 (67.6%), the mean age of the affected patients in our study was 48.61 years and the left breast was involved in 51.8%. there were no significant correlation between the Ki67 expression and the age and site of involvement in this study (P values 0.122 and 0.405) respectively, many studies also found no correlations with these parameters which support our data [1].

About 85.6% of the patients involved in this study had no recurrence or metastatic disease, and there was no any significant correlation between the recurrence or metastasis and the Ki67 in our study (P value 0.476), other studies had similar findings. It is suggested by some authors that the levels of Ki67 expression must be scores and the neoadjuvant and adjuvant chemotherapy should be modified based on the degree of expression of this tumor marked [1,13,21].

The histopathological grades of breast cancer is divided into 3 grades based on the mitotic rate and cell differentiation, in our study 50% of the patients had intermediate grade and the Ki67 was positively correlated with the grade of the tumor (P value 0.001), this correlation is concluded in some other similar articles which had the similar correlation [2].

The correlation was also positive with ER receptor in our study (P value 0.002) while was not significant with PR and HER-2 (P values 0.892 and 0.418) respectively, in some other articles the correlation was found positive also with PR [2,13].

In 70.1% of the patients involved in this study the axillary lymph nodes were involved by malignancy, but the correlation with Ki67 was not statistically significant (P value 0.971). The new guidelines don't recommend withholding adjuvant chemotherapy in patients with ER positive and low Ki67 breasct cancer patients [22,23].

The clinical stage of the tumor is one of the most important indicators for the prognosis, we adopt the TNM staging system in our classification, the majority of our cases were in the IIB stage (25.5%), followed by IIIA (22.3%), there were no statistical association between the KI67 and the stage of the disease (P value 0.509). the degree of the tumor necrosis reflects higher metabolic activity and more rapid growth and probably more aggressive biological behavior. In our study more than half of patients with available information had tumor necrosis, the correlation between necrosis and Ki67 was significant in our patients (P value 0.047) indicating that ki67 is associated with higher mitotic activity inside the tumor tissue, although little information are present in literature regarding this correlation but this finding support articles that found positive correlation with markers of invasiveness [4,9].

The main limitations of this work is that longer period of follow up in terms of duration of survival will better support the aim of this study and larger population when included will give more accurate correlations.

## 7 Conclusion

Most patients with breast cancer have positive expression of Ki67 which has a positive correlation with tumor grade, the presense of necrosis inside the tumor and estrogene receptor status. This marker is directly related with higher degrees of umor agressiveness and may be useful in modulating different treatment modalities.

## Ethical Approval

NA.

## Sources of funding

No source of funding other than the authors.

## Author contribution

Study design, data collection and analysis, writing and final approval of the manuscript: Dr Mowafak Masoud Bahaddin.

## Research registration unique identifying number (UIN)

Researchregistry**6041.**

## Guarantor

Dr Mowafak Masoud Bahaddin.

## Funding

The author was only financial supporter of the study.

## Provenance and peer review

Not commissioned, externally peer reviewed.

## Declaration of competing interest

There is no conflict of interest to be declared.

## References

[1] Mohammed A.A. (2019). Quantitative assessment of Ki67 expression in correlation with various breast cancer characteristics and survival rate; cross sectional study. Annals of Medicine and Surgery.

[2] Mahdi A.S., Ibrahim H.H., Mohammed A.A. (2018). Ki-67 expression as an indicator of invasiveness in patients with breast cancer. Med. J. Babylon.

[3] Brown J.R. (2014). Quantitative assessment Ki-67 score for prediction of response to neoadjuvant chemotherapy in breast cancer. Lab. Invest..

[4] Ragab H.M. (2018). Assessment of Ki-67 as a potential biomarker in patients with breast cancer. J. Genet. Eng. Biotechnol..

[5] Ermiah E. (2012). Prognostic value of proliferation markers: immunohistochemical ki-67 expression and cytometric s-phase fraction of women with breast cancer in Libya. J. Canc..

[6] Dowsett M. (2011). Assessment of Ki67 in breast cancer: recommendations from the international Ki67 in breast cancer working group. J. Natl. Cancer Inst..

[7] Petrelli F. (2015). Prognostic value of different cut-off levels of Ki-67 in breast cancer: a systematic review and meta-analysis of 64,196 patients. Breast Canc. Res. Treat..

[8] Klintman M. (2010). The prognostic value of Ki67 is dependent on estrogen receptor status and histological grade in premenopausal patients with node-negative breast cancer. Mod. Pathol..

[9] Stathopoulos G.P. (2014). The role of Ki-67 in the proliferation and prognosis of breast cancer molecular classification subtypes. Anti Canc. Drugs.

[10] Urruticoechea A., Smith I.E., Dowsett M. (2005). Proliferation marker Ki-67 in early breast cancer. J. Clin. Oncol..

[11] Yerushalmi R. (2010). Ki67 in breast cancer: prognostic and predictive potential. Lancet Oncol..

[12] Pathmanathan N., Balleine R.L. (2013). Ki67 and proliferation in breast cancer. J. Clin. Pathol..

[13] De Azambuja E. (2007). Ki-67 as prognostic marker in early breast cancer: a meta-analysis of published studies involving 12 155 patients. Br. J. Canc..

[14] Agha R.A. (2017). The STROCSS statement: strengthening the reporting of cohort studies in surgery. Int. J. Surg..

[15] Cheang M.C. (2009). Ki67 index, HER2 status, and prognosis of patients with luminal B breast cancer. JNCI: J. Natl. Cancer Inst..

[16] Mohammed A.A. (2020). Evaluation of mastalgia in patients presented to the breast clinic in Duhok city: cross sectional study. Ann. Med. Surg..

[17] Mohammed A.A. (2019). Accessory nipple over the right scapula of a 14-year-old boy: an extremely rare and unreported location, case report. Int. J. Surg. Case Rep..

[18] Kontzoglou K. (2013). Correlation between Ki67 and breast cancer prognosis. Oncology.

[19] Nishimura R. (2010). Ki-67 as a prognostic marker according to breast cancer subtype and a predictor of recurrence time in primary breast cancer. Exp. Therapeut. Med..

[20] Kim K.I. (2014). Ki-67 as a predictor of response to neoadjuvant chemotherapy in breast cancer patients. J. Breast canc..

[21] Jones R.L. (2009). The prognostic significance of Ki67 before and after neoadjuvant chemotherapy in breast cancer. Breast Canc. Res. Treat..

[22] Mohammed A.A. (2019). Predictive factors affecting axillary lymph node involvement in patients with breast cancer in Duhok: cross-sectional study. Annals of Medicine and Surgery.

[23] Andre F. (2015). Ki67—no evidence for its use in node-positive breast cancer. Nat. Rev. Clin. Oncol..

